# Conclusion of diagnostic odysseys due to inversions disrupting *GLI3* and *FBN1*

**DOI:** 10.1136/jmg-2022-108753

**Published:** 2022-11-21

**Authors:** Alistair T Pagnamenta, Jing Yu, Julie Evans, Philip Twiss, Genomics England Research Consortium, Musculoskeletal GeCIP MDT, Amaka C Offiah, Mohamed Wafik, Sarju G Mehta, Mohammed K Javaid, Sarah F Smithson, Jenny C Taylor

**Affiliations:** 1 Wellcome Centre for Human Genetics, University of Oxford, Oxford, Oxfordshire, UK; 2 Oxford NIHR Biomedical Research Centre, University of Oxford, Oxford, Oxfordshire, UK; 3 Nuffield Department of Clinical Neurosciences, University of Oxford, Oxford, Oxfordshire, UK; 4 Bristol Genetics Laboratory, North Bristol NHS Trust, Bristol, UK; 5 Cambridge Genomics Laboratory, Cambridge University Hospitals NHS Foundation Trust, Cambridge, Cambridgeshire, UK; 6 Academic Unit of Child Health, The University of Sheffield, Sheffield, UK; 7 Department of Clinical Genetics, Guy's and St Thomas' NHS Foundation Trust, London, UK; 8 Department of Clinical Genetics, Cambridge University Hospitals NHS Foundation Trust, Cambridge, Cambridgeshire, UK; 9 Nuffield Department of Orthopaedics, Rheumatology and Orthopaedic Sciences, University of Oxford, Oxford, Oxfordshire, UK; 10 Department of Clinical Genetics, University Hospitals Bristol NHS Foundation Trust, Bristol, UK

**Keywords:** Sequence Inversion, Molecular Diagnostic Techniques, Gene Rearrangement, Genomics, Musculoskeletal Diseases

## Abstract

Many genetic testing methodologies are biased towards picking up structural variants (SVs) that alter copy number. Copy-neutral rearrangements such as inversions are therefore likely to suffer from underascertainment. In this study, manual review prompted by a virtual multidisciplinary team meeting and subsequent bioinformatic prioritisation of data from the 100K Genomes Project was performed across 43 genes linked to well-characterised skeletal disorders. Ten individuals from three independent families were found to harbour diagnostic inversions. In two families, inverted segments of 1.2/14.8 Mb unequivocally disrupted *GLI3* and segregated with skeletal features consistent with Greig cephalopolysyndactyly syndrome. For one family, phenotypic blending was due to the opposing breakpoint lying ~45 kb from *HOXA13*. In the third family, long suspected to have Marfan syndrome, a 2.0 Mb inversion disrupting *FBN1* was identified. These findings resolved lengthy diagnostic odysseys of 9–20 years and highlight the importance of direct interaction between clinicians and data-analysts. These exemplars of a rare mutational class inform future SV prioritisation strategies within the NHS Genomic Medicine Service and similar genome sequencing initiatives. In over 30 years since these two disease-gene associations were identified, large inversions have yet to be described and so our results extend the mutational spectra linked to these conditions.

## Introduction

The rare-disease pilot phase of the 100K Genomes Project (100KGP) involved 2183 families spread across 20 different diagnostic categories.^1^ Building on previous studies,^2^ this has been a major step towards embedding whole-genome sequencing (WGS) into standard healthcare, providing valuable lessons which are being applied in the UK National Health Service (NHS) Genomic Medicine Service. One notable finding was the significant uplift in diagnostic yield made with the help of researchers, which increased the overall yield to 25%. These researcher-enabled findings included 22 non-coding variants, many of which were confirmed experimentally by splicing/luciferase studies, and several repeat expansions.

Many individuals recruited to 100KGP had previously been pre-screened by microarrays, PCR-Sanger, multiplex ligation-dependent probe amplification, exome sequencing or panel-NGS. These types of genetic analysis are typically inefficient at picking up inversions. Although traditional karyotyping can identify inversions, in most cases this is limited to events of >10 Mb and such methods are nowadays employed infrequently as a first-line test.^3^ While the latest optical mapping methods demonstrate a high concordance with traditional approaches^4^ and have the potential to be used as first-line test for detecting cryptic SVs,^5^ these methods are not yet performed routinely in clinical laboratories. Therefore, one might anticipate 100KGP to be enriched for cryptic structural variants (SVs). Given that the raison d’être of WGS is to pick up all forms of variation, the absence of diagnostic inversions or other complex copy-neutral rearrangements in the 100KGP pilot is notable. Of the 40 variants classed as SVs, all were simple deletions/duplications.^1^

This study was prompted by an unanticipated finding resulting from a virtual multidisciplinary (MDT) meeting involving clinical and academic centres in the UK set up to review genetic/clinical data for unsolved musculoskeletal cases from the 100KGP. These meetings aimed to integrate phenotypic information with dREAMS radiological characterisation^6^ and combine with manual review of genomic data. To follow-up our initial findings, which included a family with an inversion disrupting *GLI3*, bioinformatic SV prioritisation tools were developed to search systematically for gene-disrupting inversions across 43 genes that have been linked to well-characterised autosomal dominant forms of skeletal disorders.

## Methods

The 100KGP was initiated in 2013 to establish diagnoses for patients with rare-disease and cancer and promote the use of WGS in the NHS.^7^ The clinical filtering pipeline designed by Genomics England to analyse data from the 100KGP uses a tiering system (online supplemental figure S1A). Variants are assigned as tier 1–3 depending on inheritance, consequence and on whether they lie in a gene assessed as Green in PanelApp (https://panelapp.genomicsengland.co.uk), a crowdsourcing knowledgebase containing virtual gene panels relating to a wide range of human disorders. Data from the 100KGP are held in the National Genomic Research Library (https://doi.org/10.6084/m9.figshare.4530893.v6) and researchers can apply to access data at www.genomicsengland.co.uk/join-a-gecip-domain. If researchers discover variants that could represent a diagnosis for a participant, they are asked to submit the variants into a review/triage pipeline (online supplemental figure S1B), helping provide assurance to the Genomic Medicine Service that the diagnoses are of high quality and clinical relevance.

10.1136/jmg-2022-108753.supp1 Supplementary data

In the majority of rare-disease cases, DNA was extracted from blood using the EDTA method and TruSeq PCR-free high throughput library preparation was followed by 150 bp paired-read sequencing on a HiSeqX machine (Illumina). SVs were called using a combination of CANVAS and MANTA algorithms and combined into single ‘SV.vcf’ files. Mean sequence coverage for the 10 individuals reported here was 35–55 x and 341–519 inversions were called, consistent with the numbers seen across the 100KGP as a whole (mean 427; online supplemental table S1). Further quality control statistics are available within the Genomics England research environment.

A monthly virtual MDT meeting process was initiated to scrutinise clinical/WGS data with the aim of helping to solve unsolved musculoskeletal cases from the 100KGP. Further details describing these meetings are available in online supplemental methods. Manual review of read alignments was performed using IGV (v2.11.9), with visibility range threshold setting increased to 100 kb. The SV.vcf file was also loaded into IGV with the feature visibility window size set to 0 kb.

SVs are thought to play a significant role in dominant disease and yet are often missed by WGS analytical pipelines. We therefore sought to extend the preliminary results arising from the MDT meetings by focussing on 43 autosomal genes (online supplemental table S2) listed in the 2019 revision of the skeletal disorder nosology^8^ which curators at the Clinical Genome Resource (www.clinicalgenome.org) assessed as having ‘sufficient evidence’ supporting haploinsufficiency as a disease mechanism (HI=3). Gene-oriented filtering of SVs in rare disease cases from the main-programme of the 100KGP was performed with SVRare,^9^ as described in online supplemental methods. To validate inversions, breakpoint PCR and Sanger sequencing was performed using primers listed in online supplemental table S3.

## Results

**Figure 1:**
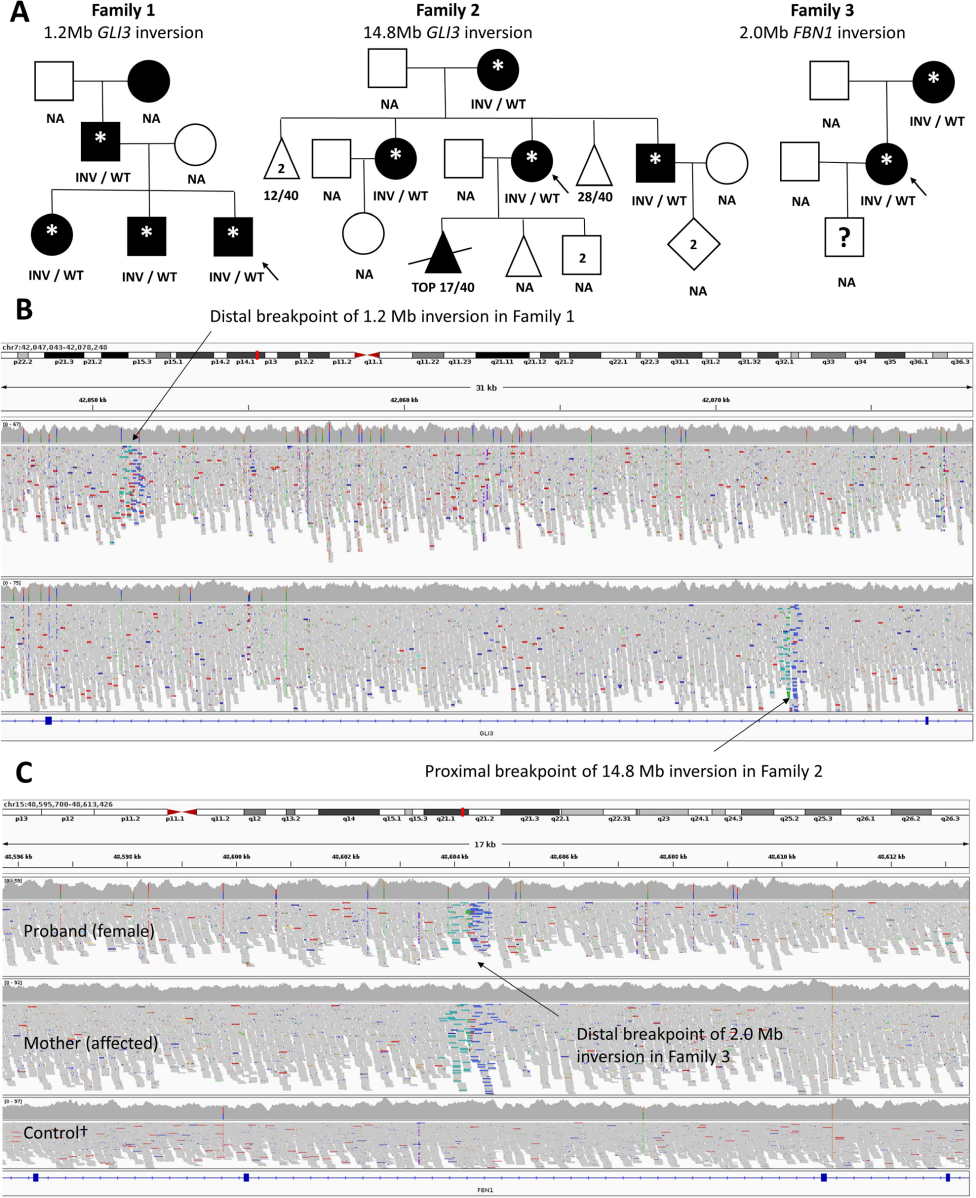
Pedigrees and characteristic read-alignment signatures for rare diagnostic inversions in three Families from 100KGP. (A) Pedigrees and genetic segregation. Shading in Family 1 indicates polysyndactyly of hands/feet, relative macrocephaly and suspected Greig syndrome. Shading in Family 2 indicates radial dysplasia, toe syndactyly and variable urogenital features, as detailed in online supplemental figure S13. Shading in Family 3 indicates thoracic aortic aneurysm and suspected Marfan syndrome. Clinical status of the proband’s son is unknown. *WGS data available from 100KGP. NA, genetic testing not performed. (B) Read-alignments viewed with IGV showing inversions of chr7:42 051 297–43 254 780 (Family 1) and chr7:27 245 456–42 072 394 (Family 2). Both *GLI3*-disrupting inversions have breakpoints in intron 4, confirming that truncation of the gene at this point is a *bona fide* disease mechanism. (C)Distal breakpoint of inversion (chr15:46 635 052–48 604 302) disrupting *FBN1* shared by proband (upper track) and mother (middle). †Control (lower) is unrelated individual from 100KGP analysed using similar methods. GRCh38 read-alignments are coloured by pair orientation such that read-pairs where both reads map to the +ve genomic strand are highlighted in green. Read-pairs where both reads map to the –ve strand (blue) are seen on the other side of the breakpoint.

Prior to the first MDT meeting, details were circulated of a boy with clinical features consistent with Greig cephalopolysyndactyly syndrome (GCS) that included relative macrocephaly, hypertelorism, postaxial polysyndactyly of hands and preaxial polysyndactyly of feet (Family 1; online supplemental figure S2). Similarly affected family members included two older siblings, the father and the paternal grandmother (figure 1A). Targeted *GLI3* sequencing in 2004 and again in 2015 had been negative (online supplemental table S4). Due to a confident clinical diagnosis of GCS syndrome, manual inspection of read alignments was performed and in 4/4 affected family members clustering of split read-pairs was identified in intron 4 (figure 1B). Relative strand orientations were consistent with the presence of a 1.2 Mb inversion. This inversion had been called by Manta as chr7:42 051 297–43 254 780 (GRCh38). While the distal breakpoint disrupts *GLI3*, the proximal breakpoint lies within *HECW1*, another gene predicted to be constrained against loss of function variants (pLI=1, gnomAD 2.1.1) but not yet associated with any Mendelian disease. Breakpoints called by Manta were consistent with those seen in the Sanger validation data (online supplemental figure S3), confirming a small ~25 bp deletion at one end (online supplemental figure S4). The genuine 1.2 Mb *GLI3* inversion lay within a larger 11.6 Mb inversion call. Manual scrutiny of read alignments suggested the latter to be an artefact and increased confidence for genuine inversions may be achieved by the fact that breakpoints are detected separately and represented twice in the SV.vcf file in a reciprocal manner (online supplemental figure S5, table S4).

**Figure 2:**
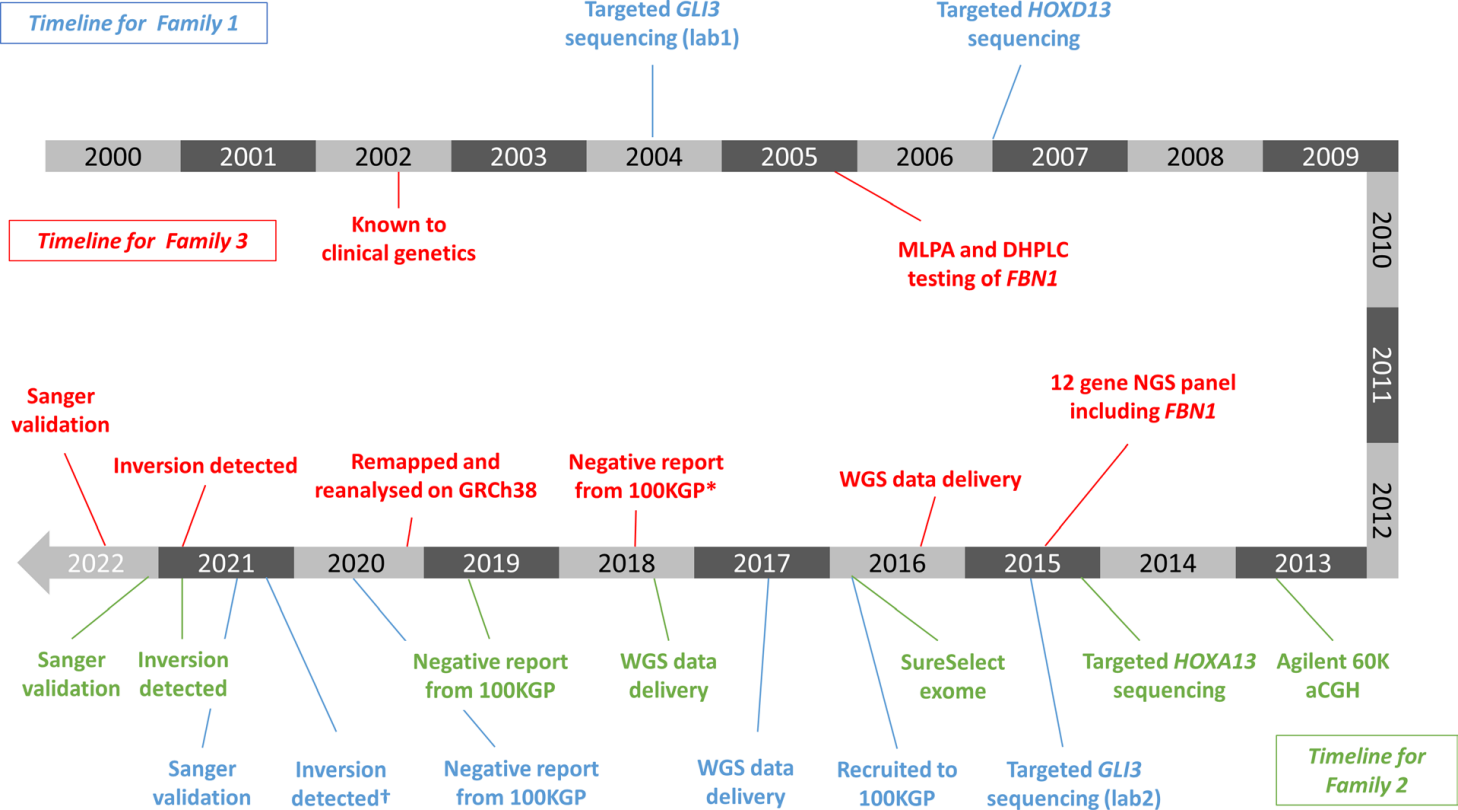
Diagnostic odyssey timelines for Families 1–3. For Family 1, precise dates were unavailable for karyotyping and array testing. *Sequence data initially analysed in 2016 using GRCh37 as a reference. The same data were remapped and reanalysed on GRCh38 in February 2020. †Variant identified on Rare Disease Day 2021. WGS, whole-genome sequencing.

As inversions are an under-reported class of SV, we sought to replicate this finding using SVRare^9^ across 71 408 rare-disease participants from 100KGP. This cohort corresponds to 33 924 families, of which 5222 were recruited under the musculoskeletal domain. Here, we focused on 43 genes linked to skeletal disorders where haploinsufficiency is a known mechanism.^8^ Although Manta typically calls ~400 inversions per genome, prioritisation is simpler than for deletions/duplications because only genes overlapping breakpoints are unequivocally disrupted. More detailed information of the filtering/interpretation process is provided in online supplemental figures S6 and S7.

Our systematic prioritisation uncovered Family 2, where a 14.8 Mb inversion (chr7:27 245 456–42 072 394) disrupting *GLI3* was identified in 4/4 affected family members (figure 1A and B). The proband was first reviewed in the genetics clinic in her early 30 s, following a termination of pregnancy due to multiple congenital abnormalities. She presented with an unusual combination of distal limb and genitourinary tract malformations. The patient was noted to have a bicornuate uterus with solitary vagina and cervix, a unilateral duplex kidney, bilateral broad and proximally placed thumbs (online supplemental figure S8A), bilateral medial displacement of the great toe (‘sandal gap’) and bilateral 2/3 toe syndactyly (online supplemental figure S8B). Clinical details for other family members are available in the online supplemental methods. Although hand-foot-genital syndrome (MIM #140000) had been suspected, targeted *HOXA13* analysis and exome sequencing failed to identify any pathogenic variants. While disruption of *GLI3* at the proximal breakpoint likely contributes to the skeletal phenotype, the distal breakpoint in 7p15.2 lies~45 kb upstream of *HOXA13* and so positional effects may underlie the more variable urogenital anomalies. Breakpoint PCR and Sanger sequencing validated the inversion and confirmed the breakpoints to be consistent with those called by Manta (online supplemental figure S9), although with a small 14 bp insertion at the proximal end and a 6 bp deletion at the distal end (online supplemental figure S10).

Lastly, a mother-daughter duo (Family 3) with Marfan syndrome suspected for ~20 years shared a 2.0 Mb inversion (chr15:46 635 052–48 604 302) disrupting *FBN1* (figure 1A, C). The daughter, first seen in the genetics clinic in her early teens, had skeletal features typical of the condition, with an increased upper segment:lower segment ratio, positive wrist and thumb signs, striae over the knees, upper legs and lower back, mild pectus excavatum and mild scoliosis. An echocardiogram showed marked aortic root dilatation. Despite previous genetic testing of *FBN1* using a variety of methods (online supplemental table S4), the family remained without a diagnosis. Additional clinical details are available in online supplemental methods. Breakpoint PCR and Sanger sequencing validated the inversion in both affected family members and confirmed the breakpoints to be consistent with those called by Manta (online supplemental figures S11 and S12). Finding the molecular cause of disease in this family will have direct clinical utility as there are several relatives for whom we may now be able to provide accurate advice about their risks. Most notably, the proband’s son would be difficult to discharge without any molecular testing, as clinical features of Marfan syndrome are often incomplete in childhood and it can be a very variable condition even in adulthood.

## Discussion

In this study, a combination of MDT discussion, manual review and systematic bioinformatics filtering helped identify rare germline inversions involving *GLI3* and *FBN1*. In family 1, the variant was found by manual assessment of a single candidate gene, prompted by an MDT meeting, highlighting the importance of having detailed phenotypic information to guide analysis. GCS is a highly recognisable condition and we recognise that for a majority of Mendelian disorders, genetic heterogeneity would make manual assessment of read alignments impractical. We therefore performed a systematic analysis of 43 genes involving 33 924 families which identified additional pathogenic inversions disrupting *GLI3* and *FBN1*, highlighting that bioinformatic prioritisation of such variants is possible. Until now, the clinical pipeline used by Genomics England has only used SV calls from Canvas, explaining why copy-neutral changes such as these have been missed. As noted in other studies,^10^ optimisation of SV calling/prioritisation is a key area for pipeline development if the full value of clinical WGS is to be realised.

No large germline inversions have been reported for these genes previously, despite both disease-gene associations being described >30 years ago.^11 12^ A recent study identified 48 novel cases with causative variants in *GLI3* and performed a review with 314 previously reported *GLI3* variants, looking primarily for genotype-phenotype correlations—none of the variants were inversions.^13^ Searching HGMD identified two historical cases of GCS with translocation breakpoints in 7p13,^14–16^ which were critical to help pinpoint this disease gene,^12^ but no inversions. Literature searches on *FBN1* identified a CAA>TTG variant^17^ but this could be classified as a multinucleotide substitution. This variant (NM_000138.5:c.1881_1883inv, p.Cys628Asn) was also present in ClinVar, alongside two other small inversions (c.6617–9_6617-8inv and c.1875_1876inv; p.Gly626Arg), but these are all much smaller than the three inversions reported here (1.2–14.8 Mb) and likely result from different mutational processes. Another recent study assessed >373 paediatric patients with Marfan syndrome and did not identify any inversions, although the methods used may have made detection of such variants difficult.^18^ Last, the Universal Mutation Database for *FBN1* (www.umd.be/FBN1) contains information about 3077 mutations, but there were no inversions reported.

Although for all three families described, the correct clinical diagnoses had been proposed previously, the precise genetic basis had remained unexplained for 9–20 years and so no specific diagnostic or predictive/prenatal test could be offered. In each case, although multiple genetic techniques were used prior to 100KGP recruitment (figure 2), most of these methods are unable to detect copy-neutral SVs such as inversions. The exception to this is karyotyping which had been performed only for Family 1. However, in that family, the inversion was 1.2 Mb in size and thus below the detection threshold. Another striking observation is that the respective diagnostic odysseys continued, even after the WGS data had been generated and the time between the sequencing data being available and reporting of the variants ranged from 3½ to 5½ years. This lag-time highlights the difficulty in picking up *bona fide* diagnostic inversions in a national clinical WGS project and the importance of understanding the limitations of the methodology employed. In such settings, a high degree of specificity is needed due to limited knowledge regarding the pathogenic importance of copy-neutral SVs.

Both GCS and Pallister-Hall syndrome (PHS) are caused by variants in *GLI3*. Genotype-phenotype correlation studies have indicated that mutations in the N-terminal and C-terminal thirds of the gene lead to GCS whereas mutations in the middle section lead to PHS.^19^ A later study confirmed this correlation and suggested the coordinates of the central PHS specific region to be between nucleotides 1998 and 3481.^20^ Both inversions reported here for Families 1 and 2 had breakpoints in intron four and so disrupt *GLI3* after cDNA position 474 and therefore these results are largely consistent with the previously reported genotype-phenotype correlation for GCS. However, for Family 1, while disruption to *GLI3* is likely responsible for most of the clinical features seen in this family, we cannot rule out that *HECW1* disruption could be relevant with respect to some of the atypical features. For Family 2, a degree of phenotypic blending seems highly plausible given the prior suspicion for hand-foot-genital syndrome. A study from 2016 used karyotyping/WGS to characterise a homozygous 66 Mb inversion that lies 523 kb upstream of *HOXA13,* found in a patient with hand-foot-genital syndrome.^21^ Given that studies using mouse limb cells suggest that expression of HoxA genes can be controlled by enhancer elements located 5′ of the gene cluster,^22^ the authors suggested that the large pericentric inversion might dysregulate the spatial/temporal expression of *HOXA13*. Due to the large distance involved, any dysregulation would likely be less severe than for dominantly acting mutations that result in disease due to haploinsufficiency, hence leading to the recessive mode of inheritance.^21^ Here, the distal breakpoint of the inversion in Family 2 lies just 45 kb from *HOXA13* and so could potentially have a more severe effect of gene regulation.

In summary, our work stresses the need to integrate multiple SV-calling algorithms and the importance of direct interaction between clinicians and data-analysts for cases where clinical suspicion points to a particular gene. Our identification of three unrelated families harbouring inversions disrupting well-known disease genes highlights examples of a rare mutational class that had not been prioritised by Genomics England’s pipeline. Manual review prompted by a virtual MDT meeting and subsequent bioinformatic prioritisation of data can help to conclude lengthy diagnostic odysseys for the respective families.
