# Supplementary material for: Conclusion of diagnostic odysseys due to inversions disrupting GLI3 and FBN1
Source: J Med Genet. 2022 Nov 21;60(5):505–10. doi: 10.1136/jmg-2022-108753 (PMC10176330; doi:10.1136/jmg-2022-108753)
Supplement: Supplementary data [file jmg-2022-108753supp001.pdf]

SHORT REPORT

Supplemental material: Conclusion of diagnostic odysseys due to inversions disrupting *GLI3* and *FBN1*

Supplemental methods

1. Musculoskeletal MDT meetings

In March 2021 we initiated a virtual multidisciplinary team meeting (MDT) process with the aim of reviewing unsolved musculoskeletal families from the 100KGP. This MDT process is ongoing, with support from an MRC grant. Clinicians are asked to circulate background information prior to these meetings, including any suspected clinical diagnoses and sets of genes/pathways potentially involved. Details of any prior genetic testing are also requested and where appropriate, radiological images are also shared in advance via an online Image Exchange Portal. Clinicians are also asked to comment on most likely modes of inheritance. Multiple disciplines attend these monthly meetings, including clinical geneticists, adult and paediatric endocrinologists/rheumatologists (including trainees), musculoskeletal radiologists and data analysts. Even when no genetic diagnosis could be established, these meetings aimed to help make recommendations for additional clinical assessment and investigations including imaging, genes to prioritise for detailed analysis and optimum sampling of other family members for segregation analysis (e.g. the parents to complete the trio).

Our initial experiences of these meetings were presented in brief at the Bone Research Society 2021 conference[1] and an early outcome from this initiative has been the description of a novel subtype of spondylometaphyseal dysplasia (MIM #619638) due to a homozygous frameshift variant in *PRKG2*.[2]

Regular participants of the MDT meeting have included:

Dr Meena Balasubramanian (Sheffield Children's NHS Foundation Trust, Sheffield, UK), Dr Judith Bubbear (Royal National Orthopaedic Hospital NHS Trust, UK), Dr Christine Burren (University Hospitals Bristol and Weston NHS Foundation Trust, Bristol, UK), Dr Alistair Calder (Great Ormond Street Hospital for Children, London, UK), Dr Jo Fairhurst (University Hospital Southampton NHS Foundation Trust, Southampton, UK), Dr Evelien Gevers (Queen Mary University of London, London, UK), Dr David Hunt (University Hospital Southampton NHS Foundation Trust, Southampton, UK), Dr Melita Irving (Guy's and St Thomas' NHS Foundation Trust, London, UK), Dr Kassim Javaid (University of Oxford, Oxford, UK), Dr Zaineb Mohsin (University of Oxford, Oxford, UK), Prof Amaka Offiah (University of Sheffield, Sheffield, UK), Dr Alistair Pagnamenta (University of Oxford, Oxford, UK), Dr Ataf Sabir (Birmingham Women's and Children's NHS Foundation Trust, Birmingham, UK), Dr Debbie Shears (Oxford University Hospitals NHS Foundation Trust, Oxford, UK), Prof Sarah Smithson (University Hospitals Bristol NHS Foundation Trust, Bristol, UK), Dr Mohnish Suri (Nottingham University Hospitals NHS Trust, Nottingham, UK), Prof Jenny Taylor (University of Oxford, Oxford, UK), Prof Andrew Wilkie (University of Oxford, Oxford, UK) and Dr Louise Wilson (Great Ormond Street Hospital for Children, London, UK).

2. Clinical phenotype – Family 1

In advance of the first MDT meeting, details were shared of a boy (then 12 years old) with clinical features of Greig syndrome including relative macrocephaly, hypertelorism, post-axial polysyndactyly of hands and pre-axial polysyndactyly of feet (Fig. S2). Radiographs identified polysyndactyly of hands (post-axial) and feet (pre-axial) and short broad metacarpals, metatarsals and phalanges. Other family members including paternal grandmother, father and two older siblings were similarly affected (Fig. 1A, Fig. S2), suggesting an autosomal dominant mode of inheritance. Additional clinical features were observed in some family members. In the proband, these included spinal cord lipoma with dermal sinus tract and recurrent tethering of spinal cord with associated syrinx, whereas his sister had an umbilical hernia, elder brother had hypospadias and paternal grandmother, a cerebral astrocytoma. These subsidiary findings are not expected in Greig syndrome and their significance is unclear. The suspected diagnosis could not initially be confirmed molecularly as targeted sequencing analysis conducted in 2004, and repeated in 2015 by a different laboratory, had been negative (Table S4). Karyotype and array-CGH testing of the affected children was also negative.

DNA for the paternal grandmother was unavailable and so recruitment to the 100KGP was limited to the other four affected family members. Filtering of variants by Genomic England's clinical pipeline was performed as a quad family using the complete penetrance option. Gene-panels from PanelApp[3] applied were Skeletal dysplasia 1.192 (352 GREEN genes); Limb disorders 1.44 (114 GREEN genes); Rare multisystem ciliopathy disorders 1.119 (89 GREEN genes), where GREEN genes are those for which there are multiple lines of independent evidence confirming the disease-gene association. These all included *GLI3*, as a GREEN-rated candidate gene and comprised a total of 447 genes. This analysis did not yield any TIER1 or TIER2 candidate variants (loss-of-function/*de novo* or missense variants in known candidate genes for the disease, respectively) and the family was signed-off as unsolved in July 2020.

3. Clinical phenotype – Family 2

The proband, a female in her early 40s, is one of three siblings of non-consanguineous Caucasian parents (Fig. 1A). She was first reviewed in the genetics clinic following a termination of pregnancy due to multiple congenital abnormalities. Similar to other affected family members (Fig. S13), she presented with an unusual combination of distal limb and genitourinary tract malformations. A diagnosis of hand-foot-uterus syndrome (MIM #140000) was, therefore, suspected upon initial assessment. The patient was noted to have a bicornuate uterus with solitary vagina and cervix, a unilateral duplex kidney, bilateral broad and proximally placed thumbs (Fig. S8A), bilateral medial displacement of the great toe ("sandal gap"), and bilateral 2/3 toe syndactyly (Fig. S8B).

The proband's elder sister had bilateral preaxial polydactyly ("duplicated thumbs") and bilateral 2/3 toe syndactyly. No urogenital abnormalities were reported. The proband's younger brother was noted to have hypospadias, bilateral undescended testes, bilateral thumb brachydactyly, and bilateral 2-3 toe syndactyly. The proband's mother was reported to have uterine didelphys, double cervix, a longitudinal vaginal septum, bilateral broad thumbs, and bilateral 2/3 toe syndactyly. She had an obstetric history that included three miscarriages: two at 12 weeks and one at 28 weeks (Fig. 1A). All affected family members had typical neurodevelopment.

Post-mortem (PM) examination of her aborted male fetus at 17 weeks gestation showed short humeri and lower limbs, marked mandibular recession, broad thumbs showing duplication of the terminal phalanges, absent rectum and anus with narrow colo-vesical fistula, absent prostate gland, single umbilical artery, urethral agenesis with early urethral obstruction sequence including megacystis, bilateral hydroureter, hydronephrosis, and renal dysplasia. Genetic investigations on uncultured placental material (targeted QF-PCR aneuploidy test) revealed trisomy for at least the region of chromosome 15 represented by five informative markers.

Complete trisomy 15, in a non-mosaic form, would be expected to contribute to fetal demise. Nevertheless, the post mortem examination revealed specific familial phenotypic features, for example; broad duplicated thumbs and urogenital anomalies (urethral agenesis). This raised the suspicion of dual genetic diagnoses in the proband's deceased son, who might have harboured the familial 14.8Mb inversion.

The skeletal limb features observed in Family 2 are congruent with a *GLI3* disruption, namely the short broad thumbs, the preaxial polydactyly, and the 1-3 toe syndactyly. The overall phenotype including urogenital abnormalities, however, was more consistent with a *HOXA13*-related disorder. Interestingly, the 7p15.2 breakpoint of the 14.8Mb inversion lies ~45kb upstream of *HOXA13*. We hypothesize that the familial inversion contributed to the phenotype observed in Family 2 via *GLI3* disruption, in addition to a potential *HOXA13* enhancer delocalization effect.

The 100KGP clinical pipeline had yielded a negative result (Sept 2019) with TIERING having been performed prioritising variants in 120 genes made up of the following panels: Limb disorders v1.2 (114 GREEN genes; *GLI3* and *HOXA13* both GREEN), Radial dysplasia 1.6 (47 GREEN genes; *GLI3* absent, *HOXA13* GREEN) and VACTERL-like phenotypes v1.22 (16 green genes; *GLI3* listed as RED, *HOXA13* GREEN).

4. Clinical phenotype – Family 3

The proband was first seen in the genetics clinic in her early teens and has had a possible diagnosis of Marfan syndrome for many years. Skeletal features were typical of the condition, with an upper segment:lower segment (US:LS) ratio of 0.79 (normal >0.85), positive wrist and thumb signs, striae over the knees, upper legs, and lower

back, mild pectus excavatum and mild scoliosis, with hypermobility, and a high palate. She had soft, stretchy, skin. An echocardiogram performed at age 11 showed marked aortic root dilatation with a diameter of 3.68cm (>95th centile). She was started on beta-blockers, aged 13. A spinal MRI identified dural ectasia at age 18. The echocardiogram also identified mitral valve prolapse and eventually she had an aortic root replacement aged 23. Genetic testing of *FBN1* was initially performed using DHPLC and MLPA. Aortic gene panel testing via Next Generation Sequencing (12 gene panel) in 2014 also did not identify a cause. Although previous testing identified a variant of unknown significance in *FBN2* (NM_001999.4:c.976C>T, p.Pro326Ser), this was not shared by the affected mother.

Her mother had a reduced US:LS ratio of 0.72, had easy bruising, striae on her thighs, pes planus, long toes and dental overcrowding. She is myopic. An echocardiogram aged 51 was unremarkable but when reviewed aged 68 she was noted to have pectus carinatum, typical facial features, striae, reduced elbow extension, and a CT aorta identified a mildly dilated sinus of Valsalva of 4.2cm (z score=1.95), and coeliac and splenic artery aneurysms. There was no other relevant family history. Over the years, other conditions were considered but Marfan syndrome was always the primary clinical diagnosis for this family.

The clinical analysis pipeline run by Genomic England focussed on genes in the thoracic aortic aneurysm or dissection panel (v1.112) from PanelApp, which contains *FBN1* as a GREEN gene. The total number of GREEN genes that were on this panel was 31. Although the genomes were originally sequenced in 2016 and reanalysed again in 2020, no TIER1 or TIER2 variants were reported.

5. Filtering structural variants with SVRare

We systematically reviewed results of gene-oriented analyses of SVs using SVRare (17th November 2021 version).[4] SVRare was built on a MySQL database that hosted 554 million SVs from 71,408 100KGP participants from 33,924 families. Of these families, 232 (including Family 1) were recruited due to unexplained skeletal dysplasia, 24 (including Family 2) due to radial dysplasia and 664 (including Family 3) due to familial thoracic aortic aneurysm disease. For the current iteration, analysis is limited to deletions, duplications and inversions. In brief, SVRare calculates the similarity between SVs of the same type by using the fraction of the overlap (intersection) over the total length (union), and SVs are considered the same if their similarity score is higher than 80%. This enables estimation of allele frequency for rare SV prioritisation. Once clustered, variants were filtered out if they were seen in >1% of individuals. To further aid discovery of disease-causing SVs, the tool also annotates each SV for familial segregation and predicts protein-coding disruption. SVRare prioritised SVs are freely available to GeCIP members as individual gene reports in the "re_gecip/shared_allGeCIPs/JingYu-SV-query" directory within the GEL research environment.

This analysis identified 4 families with predominantly balanced inversions which the breakpoint analysis suggested would unequivocally disrupt gene function and where the phenotype was consistent with well-known known genetic conditions. This included re-identification of the 1.2Mb *GLI3* inversion in Family 1 and a 13Mb *TWIST1* inversion, published previously in a mother-son duo with craniosynostosis.[5] Two additional families were identified, as described in more detail below (Families 2 and 3).

6. Detailed summary of inversions involving *GLI3*

The SVRare report for *GLI3* identified 15 rare inversions (3.8kb – 159.3Mb) that overlapped *GLI3* (Fig. S6). Of these, review of breakpoint positions indicated that only 3 would likely lead to complete gene inactivation (i.e. at least 1 breakpoint lying within the gene). One of these inversions was the same 1.2Mb inversion already identified in Family 1. Strikingly, a second quad family (Family 2) was identified where all 4 individuals had been submitted to the 100KGP with a clinical diagnosis of radial dysplasia and all 4 harboured a 14.8Mb inversion disrupting *GLI3*. This inversion had been called by Manta as involving chr7:27,245,456-42,072,394. Like Family 1, the breakpoint for Family 2 lay in intron 4 of *GLI3* but this time it was the proximal not distal end of the rearrangement (Fig. 1B). Clinical information about Family 2 is provided above. The third inversion was a much smaller 6.6kb event that would potentially invert exon 4, however closer scrutiny of read alignments suggested this variant to be an artefact, possibly due to a rare intronic deletion lying nearby.

7. Detailed summary of inversions involving *FBN1*

There were 12 rare inversions involving *FBN1* that were prioritised by SVRare that ranged in size by 3 orders of magnitude (78.5kb – 78.2Mb). Of these, only 3 inversions had breakpoints internal to the gene and thus would be predicted to disrupt gene function (Fig. S7). One of these was a 1.97Mb inversion for which the distal breakpoint lay in intron 4 of *FBN1* (Fig. 1C). This variant was called by Manta as chr15:46,635,052-48,604,302 and detected in the proband and her affected mother, both of whom had been recruited to the 100KGP with a diagnosis of "familial thoracic aortic aneurysm disease". Clinical information about this family is provided above.

The other two inversions involving *FBN1* were present in the same family – further scrutiny of the read alignments suggested they were in fact part of a complex DUP-INV-DUP structural variant (Fig. S14). The two inversions (from Manta) and two duplicated segments (from Canvas) could be explained by at least two different genetic conformations. However, neither conformation would alter gene dosage in terms of the number of complete copies of *FBN1*. Unaltered dosage would be consistent with the fact that individuals in this family did not exhibit a Marfan-like phenotype. This highlights that caution must be taken with DUP-INV-DUP type variants because with short read data there is often ambiguity regarding precisely how the genomic segments are organised and these type of rearrangement can have no effect on gene-dosage.

8. Supplemental discussion

This study commenced with MDT-based review of Family 1, a family for whom Genomics England's clinical pipeline had assessed SNVs and indels in 447 genes. From information shared prior to our MDT meeting it quickly became apparent that the clinician's initial interest was to double-check just 1 or 2 genes really thoroughly. Prior to 100KGP, targeted sequencing of *GLI3* had been done not once but twice, demonstrating the high level of suspicion for *GLI3* being involved for this family. This long diagnostic odyssey was solved by manually reviewing read alignments in IGV. We provide IGV screenshots showing the characteristic read alignment signature associated with balanced inversions (Fig. 1B,C) and hope this may prompt clinical scientists to scrutinise other unsolved cases with WGS data where there is a strong clinical suspicion pointing to a single gene.

We then developed a robust pipeline for prioritisation of rare high-confidence inversions and used this to focus on 43 genes linked to skeletal disorders, which allowed us to identify 2 more families with rare inversions. By coincidence, for all 3 inversions the breakpoint of interest lay in intron 4 and thus are highly likely to result in loss of function as it is very hard to see how gene-function, in cases with such a rearrangement so early on in a gene, could be retained.

One limitation of our follow up analysis is that it involved a manual review step and thus not every inversion was scrutinised with an equal degree of attention. In addition, inversions can often impact on gene expression via a positional effects and our prioritisation strategy may have missed such cases. Positional effects are exemplified in Family 2 where the distal breakpoint lies just 45kb upstream of *HOXA13*, but the inversion was only picked up on account of the proximal breakpoint which disrupts *GLI3*. In Family 1, we also speculate that disruption to *HECW1* could potentially explain some of the atypical features seen. Together, these cases highlight the importance of considering both breakpoint regions for rare balanced inversions.

Even with WGS data, balanced inversions can only be picked up by Manta or other algorithms that use split-read information. Currently the Genomics England structural variant pipeline only utilises Canvas and even with copy number variants there is typically a 10kb limit to resolution. This study helps emphasise the importance of developing clinical SV prioritisation pathways that can integrate multiple calling algorithms.

9. The Genomics England Research Consortium

John C. Ambrose[1]; Prabhu Arumugam[1]; Roel Bevers[1]; Marta Bleda[1]; Freya Boardman-Pretty[1,2]; Christopher R. Boustred[1]; Helen Brittain[1]; Mark J. Caulfield[1,2]; Georgia C. Chan[1]; Greg Elgar[1,2]; Tom Fowler[1]; Adam Giess[1]; Angela Hamblin[1]; Shirley Henderson[1,2]; Tim J. P. Hubbard[1]; Rob Jackson[1]; Louise J. Jones[1,2]; Dalia Kasperaviciute[1,2]; Melis Kayikci[1]; Athanasios Kousathanas[1]; Lea Lahnstein[1]; Sarah E. A.

Leigh[1]; Ivonne U. S. Leong[1]; Javier F. Lopez[1]; Fiona Maleady-Crowe[1]; Meriel McEntagart[1]; Federico Minneci[1]; Loukas Moutsianas[1,2]; Michael Mueller[1,2]; Nirupa Murugaesu[1]; Anna C. Need[1,2]; Peter O'Donovan[1]; Chris A. Odhams[1]; Christine Patch[1,2]; Mariana Buongermino Pereira[1]; Daniel Perez-Gil[1]; John Pullinger[1]; Tahrima Rahim[1]; Augusto Rendon[1]; Tim Rogers[1]; Kevin Savage[1]; Kushmita Sawant[1]; Richard H. Scott[1]; Afshan Siddiq[1]; Alexander Sieghart[1]; Samuel C. Smith[1]; Alona Sosinsky[1,2]; Alexander Stuckey[1]; Mélanie Tanguy[1]; Ana Lisa Taylor Tavares[1]; Ellen R. A. Thomas[1,2]; Simon R. Thompson[1]; Arianna Tucci[1,2]; Matthew J. Welland[1]; Eleanor Williams[1]; Katarzyna Witkowska[1,2]; Suzanne M. Wood[1,2].

1. Genomics England, London, UK

2. William Harvey Research Institute, Queen Mary University of London, London, EC1M 6BQ, UK.

Tables

Table S1: Selected QC statistics for WGS data from 10 individuals from 3 families with diagnostic inversions in *GLI3* (F1, F2) or *FBN1* (F3). QC based on data mapped to GRCh38 with decoys. *originally analysed on GRCh37. †mean across all 100KGP samples is 427. The mean across these 10 samples is 418.

**ID**	**F1 proband**	**F1 brother**	**F1 sister**	**F1 father**	**F2 proband**	**F2 sister**	**F2 brother**	**F2 mother**	**F3 proband**	**F3 mother**
Delivery date (remapping)	May 2017	Aug 2017	Aug 2017	May 2017	Apr 2018	May 2018	Apr 2018	Apr 2018	Apr2016* (Feb 2020)	May2016* (Feb 2020)
Total aligned reads	871,004,459	1,052,818,353	932,959,733	755,979,204	1,025,504,334	749,957,343	884,423,031	815,883,876	816,243,785	1,281,867,717
Percent duplicate aligned reads	8.77%	9.14%	7.66%	6.78%	5.82%	6.02%	8.03%	8.52%	7.61%	14.55%
Percent aligned reads	93.19%	92.88%	92.73%	92.15%	93.98%	91.81%	92.30%	93.60%	95.15%	94.78%
Percent read pairs aligned to different chromosomes	0.17%	0.17%	0.15%	0.19%	0.31%	0.76%	1.50%	0.53%	0.71%	0.43%
Percent soft-clipped bases	2.01%:	1.82%	1.94%	1.91%	1.74%	1.42%	1.64%	1.56%	1.84%	2.02%
Mean coverage	39.64	47.78	43.02	35.18	48.31	35.36	40.67	37.39	37.75	54.83
Coverage at 15X	97.40%	98.07%	97.32%	96.50%	97.56%	97.04%	97.64%	97.22%	97.44%	97.93%
Fragment length median	493	490	473	500	448	495	442	484	437	491
SNVs (All)	3897444	3882458	3914338	3884196	3926566	3945642	3886341	3905448	3910612	3969352
SNV Het/Hom ratio	1.605	1.553	1.562	1.57	1.595	1.606	1.584	1.527	1.556	1.574
SNV Ts/Tv ratio	2.061	2.066	2.062	2.059	2.062	2.06	2.06	2.059	2.057	2.067
SNVs (Percent found in dbSNP)	94.63%	94.69%	94.75%	94.62%	94.59%	94.42%	94.54%	94.59%	94.41%	94.61%
Indels (All)	959057	971729	968234	936189	1003501	967602	977021	974545	971216	1005026
SV Inversions (All)†	406	432	379	341	481	361	400	427	432	519
SV Inversions in genes	235	251	215	176	290	203	226	246	258	310

Table S2: Details of 43 autosomal genes listed in 2019 revision of the skeletal disorder nosology[6] for which ClinGen assess as having "sufficient evidence" supporting haploinsufficiency (downloaded 10th January 2022).

Gene symbol	Approved name	HGNC ID	Location
ALX4	ALX homeobox 4	HGNC:450	11p11.2
ARID1B	AT-rich interaction domain 1B	HGNC:18040	6q25.3
CDKN1C	cyclin dependent kinase inhibitor 1C	HGNC:1786	11p15.4
COL1A1	collagen type I alpha 1 chain	HGNC:2197	17q21.33
COL2A1	collagen type II alpha 1 chain	HGNC:2200	12q13.11
CREBBP	CREB binding protein	HGNC:2348	16p13.3
EFTUD2	elongation factor Tu GTP binding domain containing 2	HGNC:30858	17q21.31
EP300	E1A binding protein p300	HGNC:3373	22q13.2
ERF	ETS2 repressor factor	HGNC:3444	19q13.2
EXT1	exostosin glycosyltransferase 1	HGNC:3512	8q24.11
EXT2	exostosin glycosyltransferase 2	HGNC:3513	11p11.2
FBN1	fibrillin 1	HGNC:3603	15q21.1
FGF10	fibroblast growth factor 10	HGNC:3666	5p12
FGFR1	fibroblast growth factor receptor 1	HGNC:3688	8p11.23
GDF5	growth differentiation factor 5	HGNC:4220	20q11.22
GLI3	GLI family zinc finger 3	HGNC:4319	7p14.1
GNAS	GNAS complex locus	HGNC:4392	20q13.32
HOXD13	homeobox D13	HGNC:5136	2q31.1
KAT6B	lysine acetyltransferase 6B	HGNC:17582	10q22.2
LEMD3	LEM domain containing 3	HGNC:28887	12q14.3
LMX1B	LIM homeobox transcription factor 1 beta	HGNC:6654	9q33.3
MNX1	motor neuron and pancreas homeobox 1	HGNC:4979	7q36.3
MYCN	MYCN proto-oncogene, bHLH transcription factor	HGNC:7559	2p24.3
NF1	neurofibromin 1	HGNC:7765	17q11.2
NIPBL	NIPBL cohesin loading factor	HGNC:28862	5p13.2
NOG	noggin	HGNC:7866	17q22
NSD1	nuclear receptor binding SET domain protein 1	HGNC:14234	5q35.3
PAX3	paired box 3	HGNC:8617	2q36.1
POLR1D	RNA polymerase I and III subunit D	HGNC:20422	13q12.2
PTPN11	protein tyrosine phosphatase non-receptor type 11	HGNC:9644	12q24.13
SALL4	spalt like transcription factor 4	HGNC:15924	20q13.2
SF3B4	splicing factor 3b subunit 4	HGNC:10771	1q21.2
SHH	sonic hedgehog signalling molecule	HGNC:10848	7q36.3
SMAD3	SMAD family member 3	HGNC:6769	15q22.33
SMAD4	SMAD family member 4	HGNC:6770	18q21.2
SMARCB1	SWI/SNF related, matrix associated, actin dependent regulator of chromatin, subfamily b, member 1	HGNC:11103	22q11.23
TBX3	T-box transcription factor 3	HGNC:11602	12q24.21
TBX4	T-box transcription factor 4	HGNC:11603	17q23.2
TBX5	T-box transcription factor 5	HGNC:11604	12q24.21
TCF12	transcription factor 12	HGNC:11623	15q21.3
TCOF1	treacle ribosome biogenesis factor 1	HGNC:11654	5q32-q33.1
TRPS1	transcriptional repressor GATA binding 1	HGNC:12340	8q23.3
TWIST1	twist family bHLH transcription factor 1	HGNC:12428	7p21.1

Table S3: Primers used for Sanger validation in Families 1, 2 and 3.

Family	Primer name	Sequence	End of inversion
Family 1	GLI3_Inv1_F_V1	TACTGCTGAGAAGCAACAGTG	Distal
	GLI3_Inv1_R_V1	CAGCTTTCTTAGATATGATATAC	
	GLI3_Inv2_F_V1	AGTATATACTAGGCTCAGTACATG	Proximal
	GLI3_Inv2_R_V1	GAAGGTTAGGGTGTATAAATGAC	
Family 2	GLI3-1F	CCGGGAGAACTACGTATCCA	Distal
	GLI3-2F	CCCTGCTTTGGAAAATGAAT	
	GLI3-1R	TGTGTGTATGGGAGGAGCAG	Proximal
	GLI3-2R	TGGGAATGTAGGCAATTGGT	
Family 3	FBN1-INV-1F	TCCCCAAGACGAAATGAACTT	Proximal
	FBN1-INV-2F	GGCACCTGGATCTCAATACCT	
	FBN1-INV-1R	CCCTCTGTGACAATGCCAAG	Distal
	FBN1-INV-2R	GTGTGTCTTTAGGCATCCCC	

Table S4: Details of diagnostic odysseys and prior genetic testing for Families 1-3.

**Family number**	**Family 1**	**Family 2**	**Family 3**
**Date variant detected**	February 2021	November 2021	November 2021
**Proband WGS date**	May 2017	April 2018	April 2016 (Feb 2020 for remapping to GRCh38)
**Negative report issued from 100KGP**	July 2020	September 2019	May 2018
**Time from WGS to variant being identified**	3¾ years	3½ years	5½ years
**Start of diagnostic odyssey**	2004	August 2013	Family known to clinical genetics since ca. 2002
**Gender (proband)**	Male	Female	Female
**Ethnicity**	White British	White British	White British
**GRCh38 coordinates from Manta (reciprocal call)**	chr7:42,051,297-43,254,780 (chr7:42,051,291-43,254,759)	chr7:27,245,456-42,072,394 (chr7:27,245,448-42,072,397)	chr15:46,635,052-48,604,302 (chr15:46,635,053-48,604,300)
**Size of inversion**	1.20Mb	14.83Mb	1.97Mb
**Position of breakpoint in gene**	Intron 4 of *GLI3* (NM_000168.6)	Intron 4 of *GLI3* (NM_000168.6)	Intron 4 of *FBN1* (NM_000138.5)
**Other breakpoint**	Intron 3 of *HECW1* (NM_015052.5)	Intron 2 of *EVX1* (NM_001989.5); ~45kb from *HOXA13*	No genes nearby
**Family structure**	Affected brother/sister/ father all in 100KGP	Affected sister/brother/mother all in 100KGP	Patient and her affected mother are in 100KGP
**Recruitment diagnosis**	Unexplained skeletal dysplasia	Radial dysplasia	Familial Thoracic Aortic Aneurysm Disease
**Cytogenetic testing**	Karyotyping done but no details available	No indication that karyotyping was ever done, but has now been requested	N/A
**Array testing (date, array type/version)**	Array CGH done but no details available	Agilent 60K aCGH (design version 028469). Median resolution: 120kb. Oct 2013	N/A
**MLPA testing**	N/A	N/A	Genetic testing of *FBN1* for large deletions and duplications by MLPA in 2005
**Targeted sequencing**	Targeted sequencing of *GLI3* first in 2004 (Biesecker lab) and then repeated in 2015 using PCR-Sanger method. *HOXD13* also sequenced in 2006-7 in Oxford	Analysis of *HOXA13* and flanking intronic sequences by PCR multiplex AmpliSeq (IAD47762_93, Ion Torrent. Also by PCR and Sanger sequencing. Done in Lille (Hopital Jeanne de Flandre) January 2015	NGS testing of *ACTA2, COL3A1, EFEMP2, FBN1, FBN2, FLNA, MYH11, MYLK, NOTCH1, SK1, SLC2A, SMAD3* in 2015, using the Illumina Trusight One sequencing panel
**Exome testing**	N/A	WES at Viapath with Agilent SureSelectXT Clinical Research Exome (SureSelectXT Human All Exon V5 baited with clinically relevant genes). The enriched exome libraries WES using paired-end, 125 cycle chemistry on an Illumina HiSeq2500. November 2016.	N/A

**Other genetic testing**	N/A	The proband's aborted fetus has had QF-PCR aneuploidy test for chromosomes 13, 14, 15, 16, 18, 21, 22, X and Y. The test showed trisomy for at least the region of chromosome 15 represented by five informative markers. The test was done on uncultured placental material and is likely the cause of the fetal loss.	Testing of *FBN1* by DHPLC in 2005
**Other variants from GEL pipeline**	No TIER1/2 from GEL pipeline	No TIER1/2 from GEL pipeline	No TIER1/2 from GEL pipeline. Previous testing identified *FBN2* variant NM_001999.4:c.976C>T (p.Pro326Ser) in proband. Variant not seen in mother and too common in gnomAD so LB/B, see www.ncbi.nlm.nih.gov/clinvar/variation/137316.
**Validation of inversion**	PCR of both inversion breakpoints and Sanger sequencing in 4/4 affected individuals - May 2021	PCR of both inversion breakpoints in 4/4 affected individuals and Sanger sequencing (proband only) – January 2022; karyotyping is underway	PCR and Sanger sequencing – May 2022
**Reason to check gene and detection method**	Clinical suspicion for *GLI3* mutation shared with data analyst prior to March 2021 MDT meeting. Detected by manual review of read alignments but also called by Manta	AD gene mentioned in Mortier *et al* 2019[6] where haploinsufficiency is a known mechanism of pathogenesis. Detection by Manta following genome sequencing in 100KGP and prioritised by SVRare[4]	

Supplemental Figures

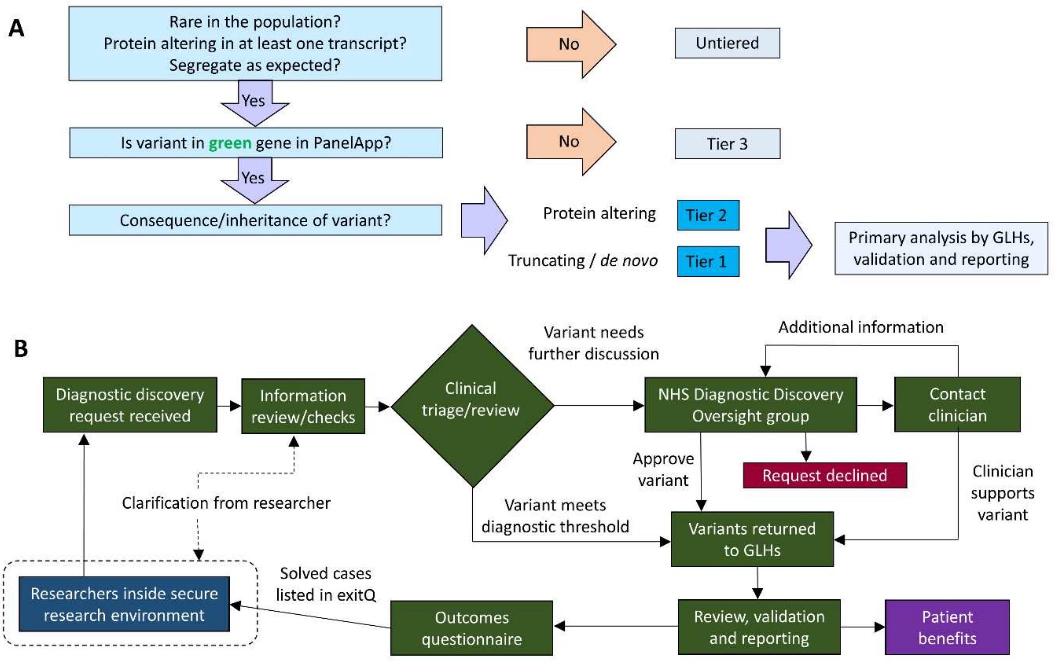

Figure S1: Summary of clinical tiering and researcher identified variant pipelines linked to the 100K Genomes Project. A) Schematic of the clinical tiering pipeline employed by Genomic England. Although only Tier 1 and 2 variants are assessed routinely by the respective Genomic Laboratory Hub (GLH), Tier 3 variants are also available for review, if required. B) Researchers may discover variants that could represent a diagnosis for a participant. This diagram summarises the official pathway to feed back these findings to the NHS Diagnostic Discovery Oversight Group. This group comprises clinicians and scientists from each GLH, whom meet every 2 months with NHS England and Genomics England representatives. This pipeline provides assurance to the Genomic Medicine Service that the diagnoses being returned are of high quality and clinical relevance. A separate pathway exists for the rapid return of variants considered to be urgent.

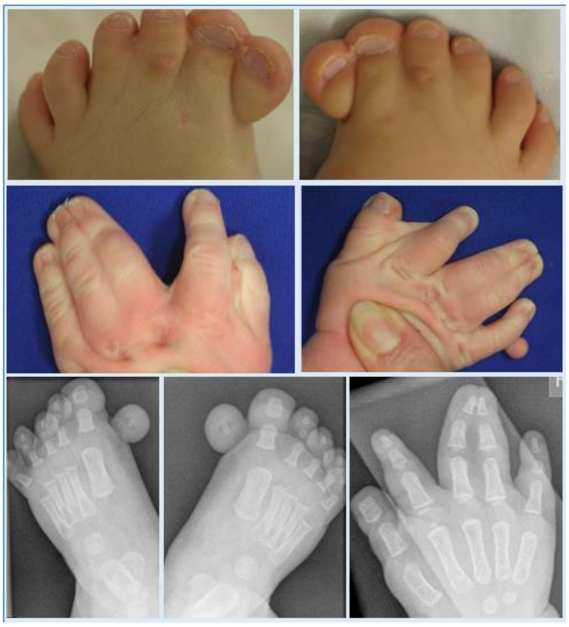

Figure S2: Distal limb anomalies observed in siblings of Family 1 are shown, including symmetrical pre-axial polysyndactyly of feet of sister (above) and post-axial polysyndactyly of hands of brother (mid-section). Radiographs of the feet of the proband (below, left and centre) illustrate the interfamilial variability of pre-axial polydactyly with a single proximal phalanx on each side. The first metatarsals are broad. Radiograph of right hand of brother (below right) shows 3-4 soft tissue and bone (terminal phalangeal) syndactyly with relatively short metacarpals and middle/terminal phalanges (post-axial additional digit previously surgically removed).

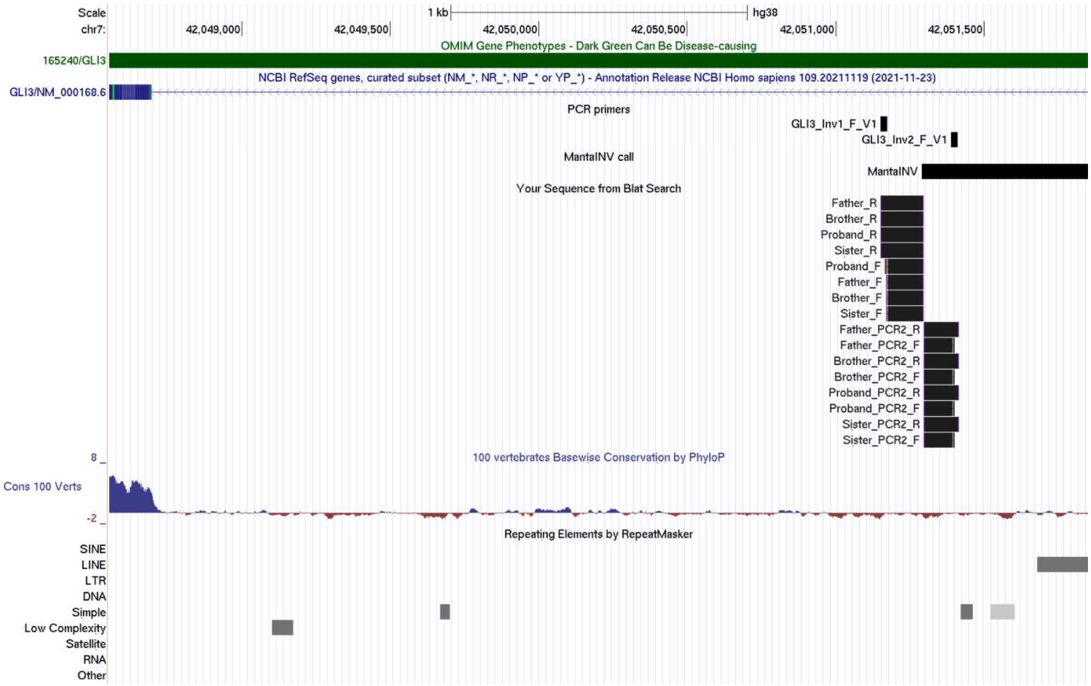

Figure S3: Sanger validation and primer positions for distal end of the *GLI3* inversion in Family 1. PCR primer positions are shown for 2 of the 4 primers. Amplicons for 4 affected family members were sequenced bidirectionally and the resulting sequences were uploaded to the UCSC genome browser in FASTA format using the Blat Search tool. An interactive view is shown at https://genome.ucsc.edu/s/AlistairP/GLI3_SANGER where one can navigate to the proximal end of the inversion and see the same pattern. The breakpoints defined by Manta are consistent with the Sanger traces.

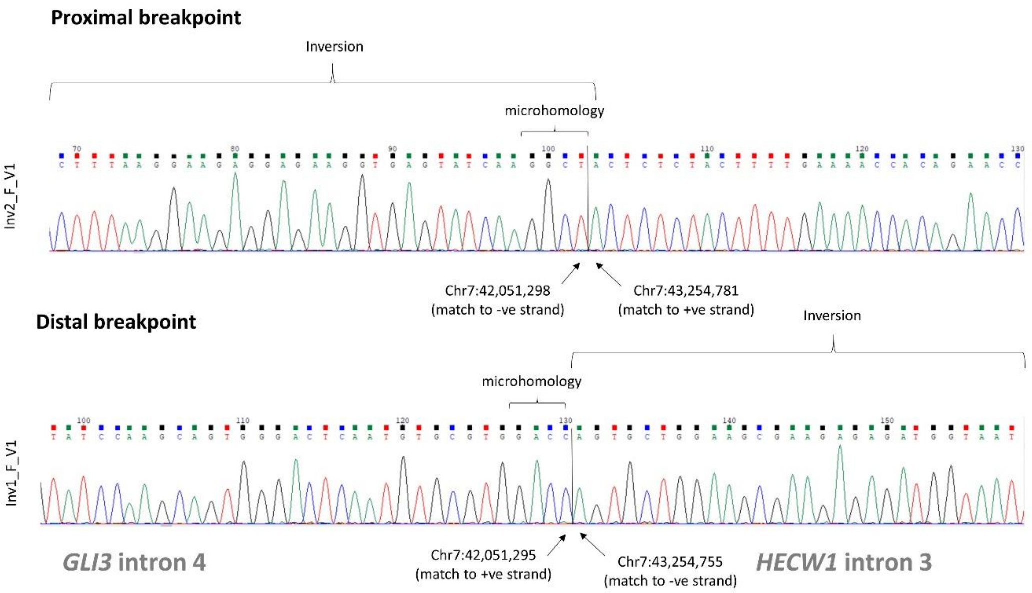

Figure S4: Sanger sequencing electropherograms confirming breakpoints for the 1.2Mb inversion in Family 1. Close scrutiny reveals a 25bp deletion at the proximal end and 4bp of microhomology at the respective junctions. Genomic positions are based on GRCh38.

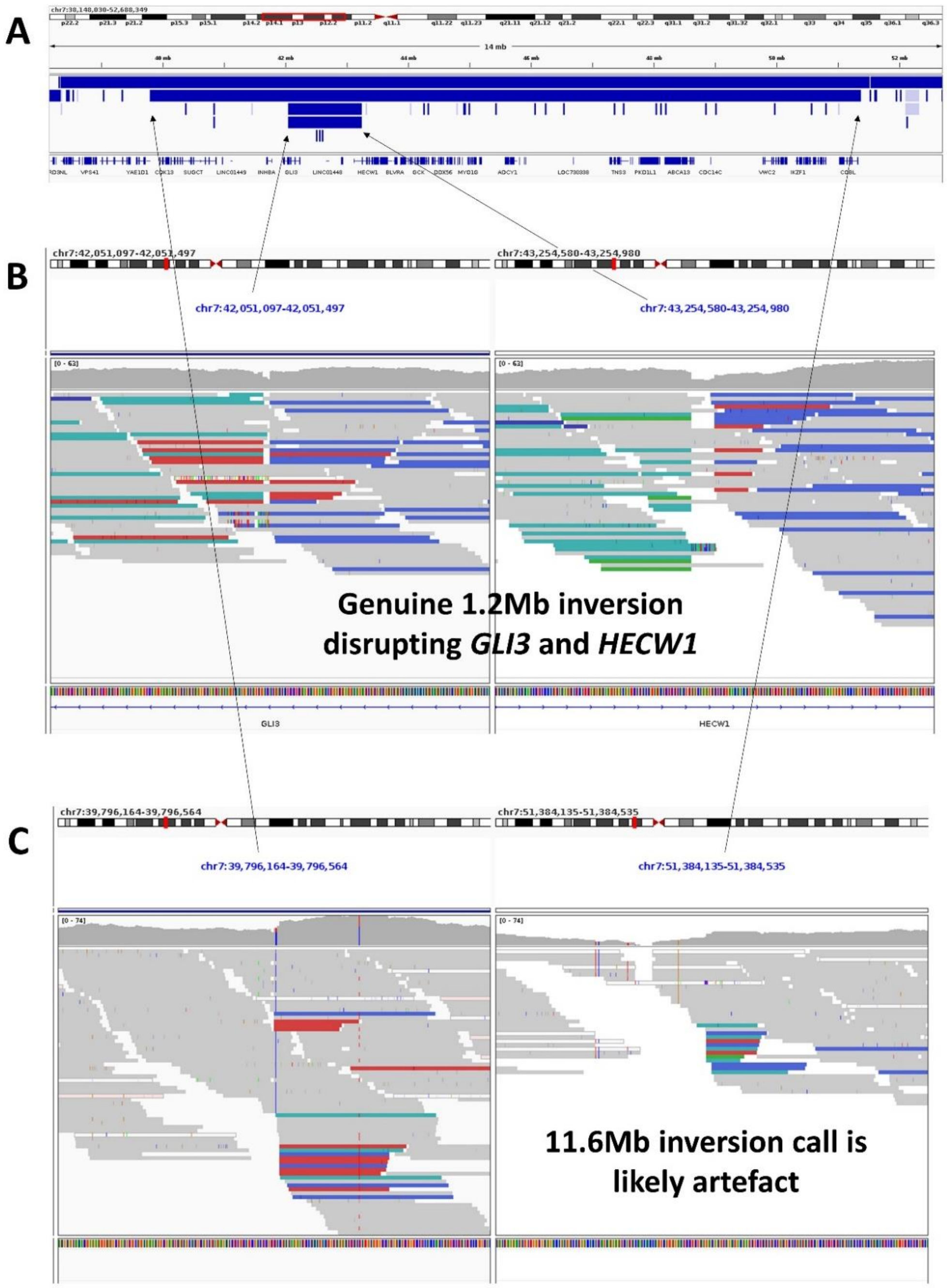

Figure S5: IGV screenshot showing Manta/Canvas SV calls and read alignments in the proband from Family 1. A) Manta calls from structural variant vcf file suggests an inversion within a larger inversion on 7p. Genuine inversions such as the 1.2Mb inversion that disrupts *GLI3* are often reported twice in the SV.vcf in a reciprocal manner. B) +ve to +ve read pairs (green) and -ve to -ve read pairs (blue) are seen on each side of the breakpoints. At this level of zoom, the small deleted region is visible at the proximal end of the 1.2Mb inversion. C) Artefactual inversion calls such as the larger 11.6Mb one shown above are only supported by one breakpoint (in this case -ve to -ve strand read pairs) and coverage is more variable.

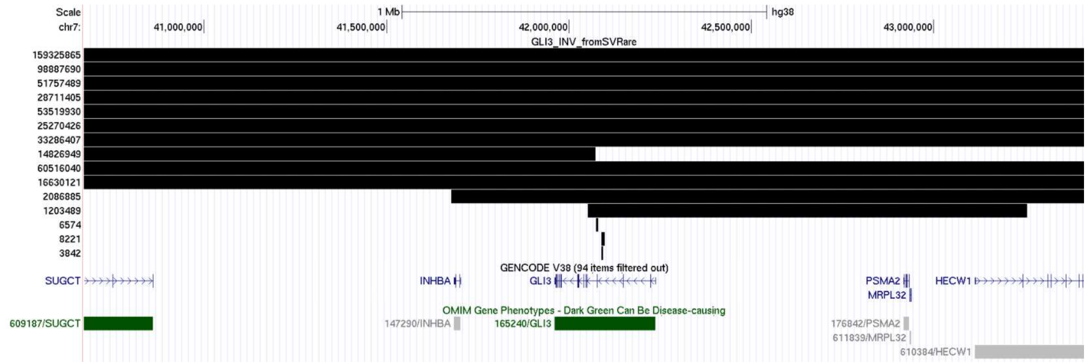

Figure S6: Screenshot of UCSC genome browser graphic showing positions of rare inversion calls overlapping *GLI3* in the rare disease arm of the 100KGP. The custom track "GLI3_INV_fromSVRare" contains 15 entries labelled by size in bp, of which only 2 large inversions directly disrupt *GLI3*. The first of these was reidentification of the 1.2Mb inversion seen in Family 1. The second was a 14.8Mb inversion in Family 2. The 6574bp event would in theory invert a single exon but review of read alignments suggested that this may be an artefact on account of a nearby intronic deletion. An interactive version is available here: https://genome.ucsc.edu/s/AlistairP/GLI3_INVERSION_F2.

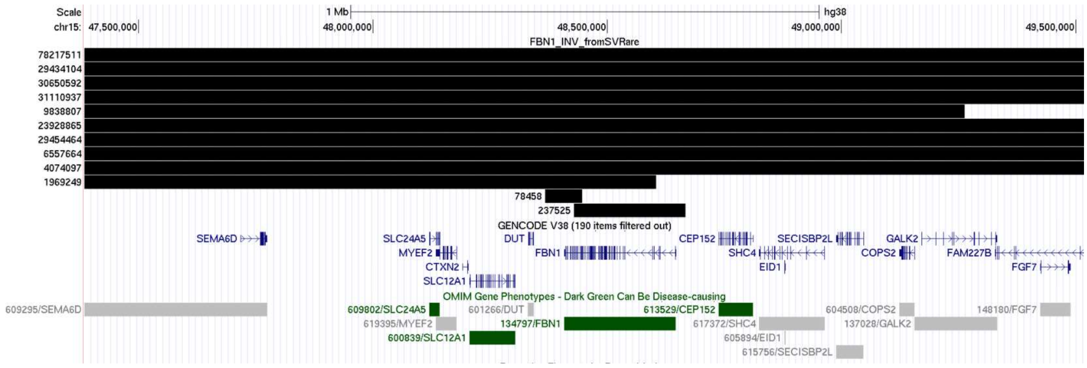

Figure S7: Screenshot of UCSC genome browser showing positions of rare inversion calls overlapping *FBN1* in the rare disease arm of the 100KGP. Only 3 of the 12 rare inversions prioritised by SVRare and shown in the track "FBN1_INV_fromSVRare" have breakpoints which disrupt *FBN1* directly. The 1.97Mb inversion identified in Family 3 likely results in loss of function. The 78.5kb and 237.5kb inversions are from the same family and appear to represent a complex DUP-INV-DUP which is inherited from an unaffected parent. An interactive version is available here: https://genome.ucsc.edu/s/AlistairP/FBN1_INV_SVRare.

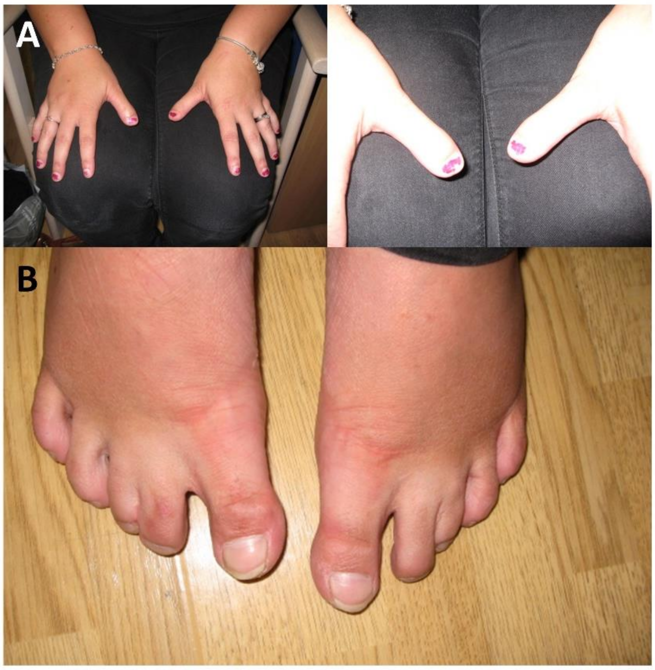

Figure S8: Clinical photographs of proband in Family 2. A) Hands showing bilateral short and broad thumbs. B) Feet displaying bilateral 2/3 toe syndactyly and sandal gap.

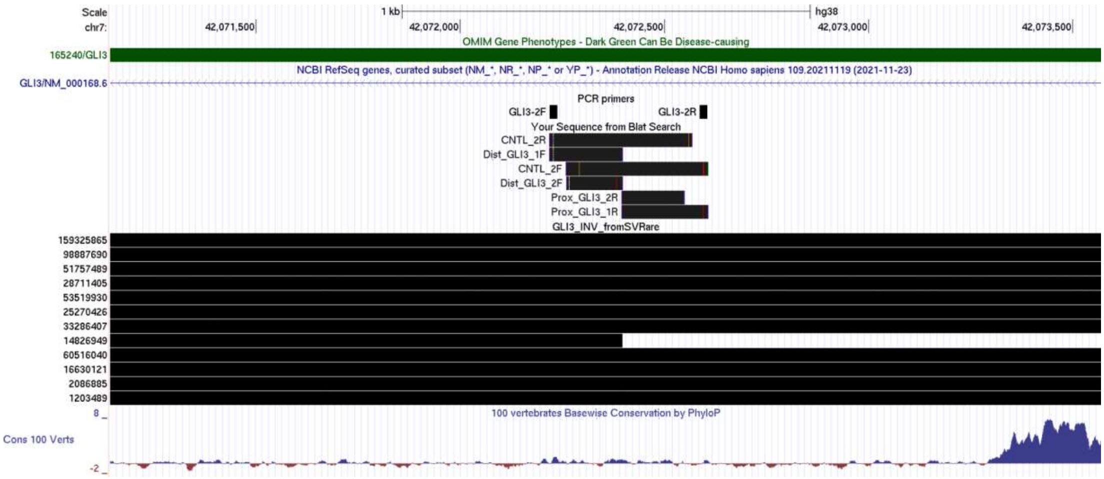

Figure S9: Sanger validation and primer positions for proximal end of the *GLI3* inversion in Family 2. PCR primer positions are shown for 2 of the 4 primers. PCR amplicons for the proband were sequenced bidirectionally and the resulting sequences were uploaded to the UCSC genome browser in FASTA format using the Blat Search tool. An interactive view is shown at https://genome.ucsc.edu/s/AlistairP/GLI3_INVERSION_F2_SANGER where one can navigate to the distal end of the inversion and see the same pattern. Breakpoints for the 14.8Mb inversion as defined by Manta are consistent with the Sanger data.

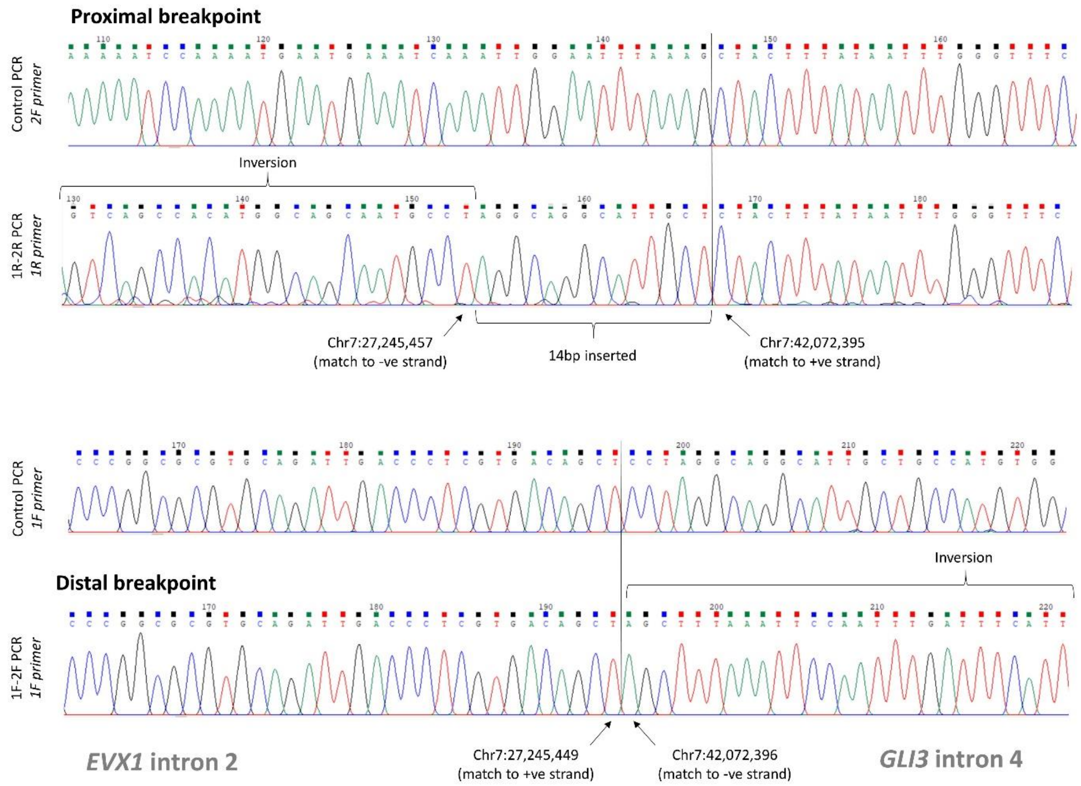

Figure S10: Sanger sequencing electropherograms confirming breakpoints for the 14.8Mb inversion in Family 2. Close scrutiny reveals a 14bp insertion at the proximal end and a 6bp deletion at the distal end. Genomic positions are based on GRCh38.

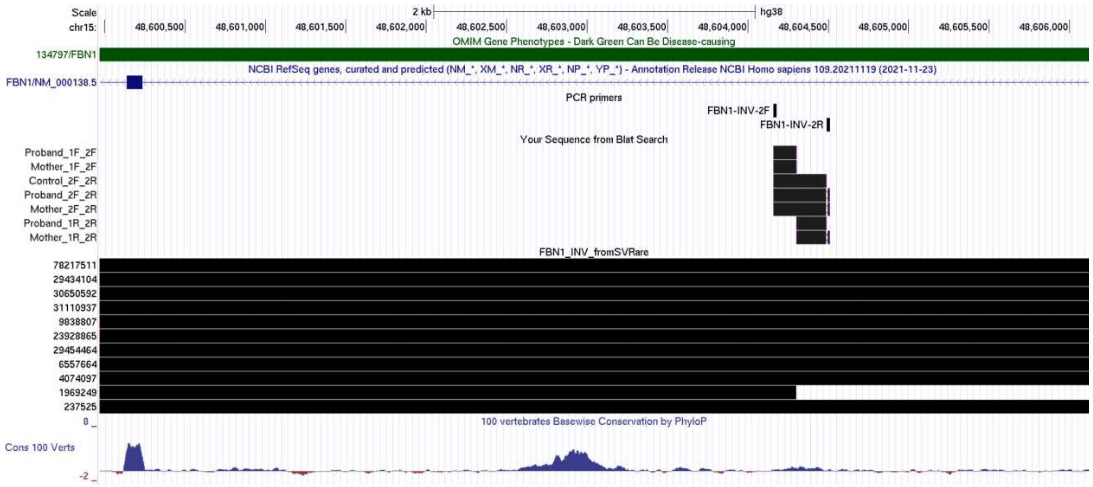

Figure S11: Sanger validation and primer positions for distal end of the *FBN1* inversion in Family 3. PCR primer positions are shown for 2/4 primers. Amplicons for the proband, mother and a control were sequenced and the resulting sequences were uploaded to the UCSC genome browser in FASTA format using the Blat Search tool. An interactive view is available at https://genome.ucsc.edu/s/AlistairP/FBN1_INVERSION_F3_SANGER where one can navigate to the proximal end of the inversion and see the same pattern. Breakpoints for the 1.97Mb inversion as defined by Manta are consistent with the Sanger data.

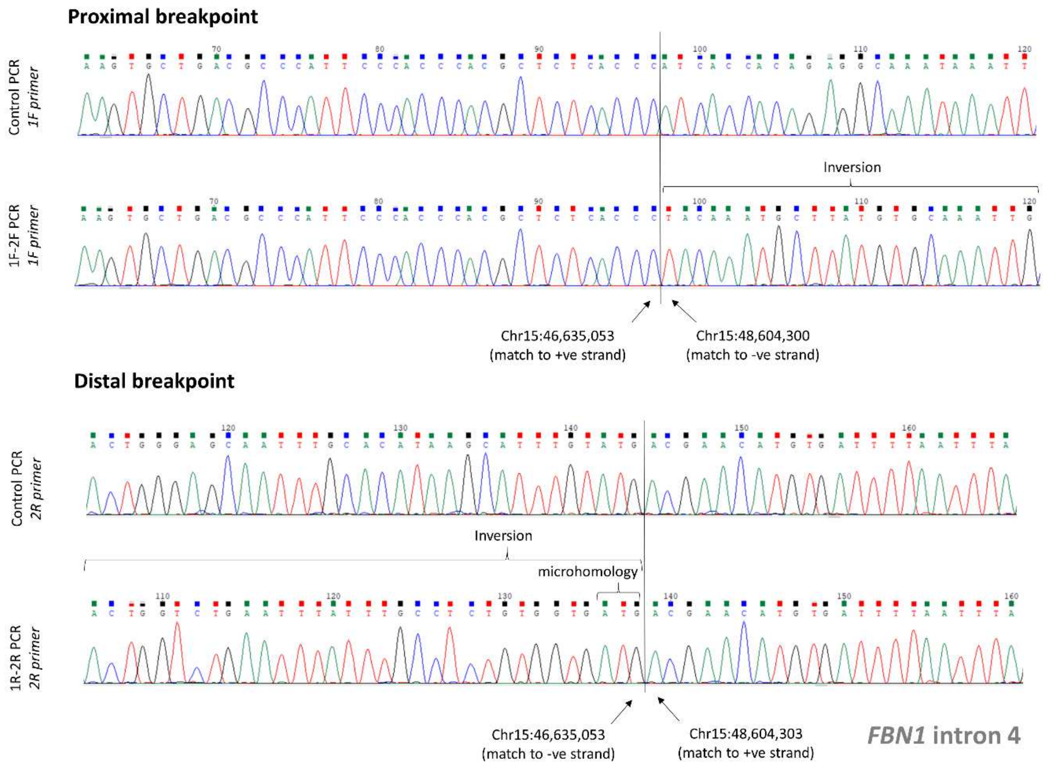

Figure S12: Sanger sequencing electropherograms confirming breakpoints for the 1.97Mb inversion in Family 3. Close scrutiny reveals 3bp of microhomology at the distal junction - the resulting ambiguity in annotation may explain why the coordinates appear to be 1bp out compared to the MantaINV call. Genomic positions are based on GRCh38.

Family 2
14.8Mb *GLI3* inversion

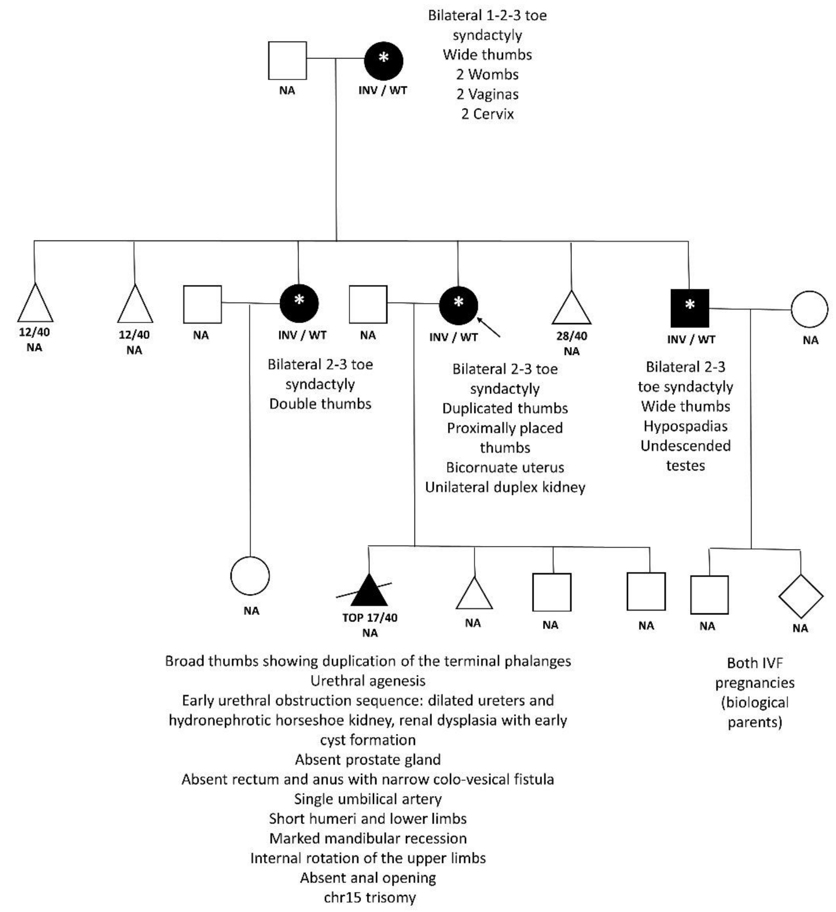

Figure S13: Detailed pedigree and additional clinical information for Family 2.

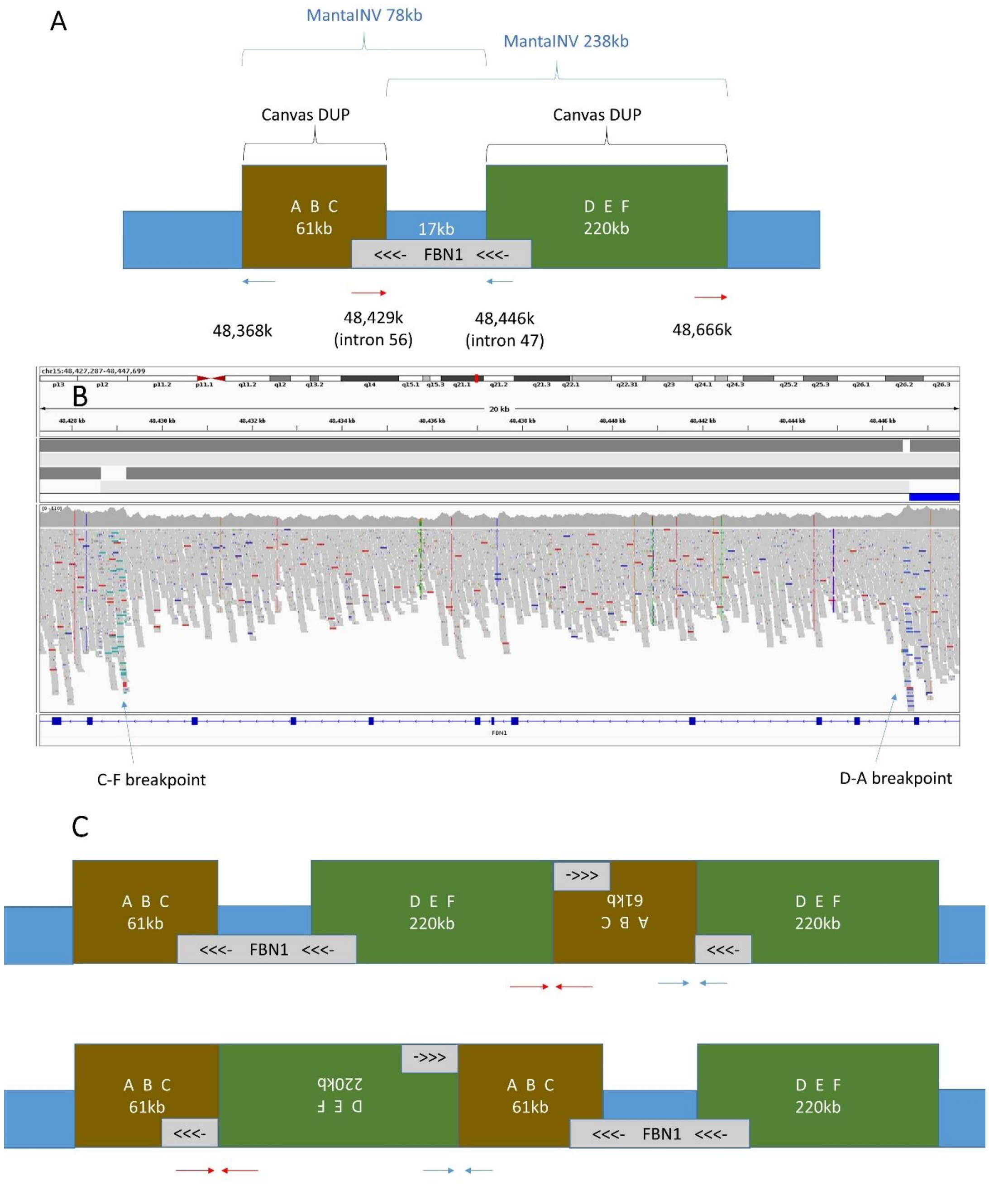

Figure S14: DUP-INV-DUP rearrangement in a 100KGP family with non-Marfan phenotype. A) Schematic diagram showing relative positions of 78kb and 238kb inversions and duplication calls with respect to *FBN1*. B) IGV screenshot showing read alignments supporting both junctions internal to *FBN1* in introns 47 and 56. C) Two possible configurations can explain the pattern of split-reads but neither are predicted to impact on dosage of full functional copies of *FBN1*.

References

1. Pagnamenta AT. Bone Research Society 2021 Abstracts - "Experiences of a virtual multidisciplinary team meetingprocess to review unsolved musculoskeletal families fromthe 100 K Genomes Project". *JBMR Plus* 2021 doi: https://doi.org/10.1002/jbm4.10552
2. Pagnamenta AT, Diaz-Gonzalez F, Banos-Pinero B, et al. Variable skeletal phenotypes associated with biallelic variants in PRKG2. *J Med Genet* 2021 doi: 10.1136/jmedgenet-2021-108027 [published Online First: 2021/11/17]
3. Martin AR, Williams E, Foulger RE, et al. PanelApp crowdsources expert knowledge to establish consensus diagnostic gene panels. *Nat Genet* 2019;51(11):1560-65. doi: 10.1038/s41588-019-0528-2 [published Online First: 2019/11/05]
4. Yu J, Szabo A, Pagnamenta AT, et al. SVRare: discovering disease-causing structural variants in the 100K Genomes Project. *medRxiv* 2022 doi: https://doi.org/10.1101/2021.10.15.21265069
5. Hyder Z, Calpena E, Pei Y, et al. Evaluating the performance of a clinical genome sequencing program for diagnosis of rare genetic disease, seen through the lens of craniosynostosis. *Genet Med* 2021;23(12):2360-68. doi: 10.1038/s41436-021-01297-5 [published Online First: 2021/08/26]
6. Mortier GR, Cohn DH, Cormier-Daire V, et al. Nosology and classification of genetic skeletal disorders: 2019 revision. *Am J Med Genet A* 2019;179(12):2393-419. doi: 10.1002/ajmg.a.61366 [published Online First: 2019/10/22]
